# Advances in Psychotropic Treatment for Pregnant Women: Efficacy, Adverse Outcomes, and Therapeutic Monitoring

**DOI:** 10.3390/jcm13154398

**Published:** 2024-07-27

**Authors:** Bárbara Costa, Nuno Vale

**Affiliations:** 1 PerMed Research Group, Center for Health Technology and Services Research (CINTESIS), Rua Doutor Plácido da Costa, 4200-450 Porto, Portugal; b.c.211297@gmail.com; 2 CINTESIS@RISE, Faculty of Medicine, University of Porto, Alameda Professor Hernâni Monteiro, 4200-319 Porto, Portugal; 3 Department of Community Medicine, Health Information and Decision (MEDCIDS), Faculty of Medicine, University of Porto, Alameda Professor Hernâni Monteiro, 4200-319 Porto, Portugal; 4 Centre for Parasite Biology and Immunology, Department of Infectious Diseases, National Health Institute Dr. Ricardo Jorge, Rua Alexandre Herculano 321, 4000-055 Porto, Portugal

**Keywords:** psychotropic therapy, pregnant women, maternal mental health, pharmacokinetics, personalized dosing, therapeutic monitoring, adverse outcomes

## Abstract

Advancements in psychotropic therapy for pregnant women are pivotal for addressing maternal mental health during the perinatal period. Screening for mood and anxiety symptoms during pregnancy is recommended to enable early intervention. Psychotropic medications, including antidepressants, benzodiazepines, antipsychotics, and mood stabilizers, are commonly used, but challenges remain regarding their safety and efficacy during pregnancy. Pregnancy induces significant changes in pharmacokinetics, necessitating personalized dosing strategies and careful monitoring. Real-time monitoring technologies, such as smartphone-integrated platforms and home-based monitoring, enhance accessibility and accuracy. Prospective studies and collaboration among healthcare providers are essential for evidence-based guidelines and optimal treatment strategies. Reducing stigma around mental health during pregnancy is crucial to ensure women seek help and discuss treatment options, promoting understanding and acceptance within the community.

## 1. Current State of Perinatal Psychiatric Disorders

Perinatal psychiatric disorders, encompassing mental health conditions occurring during pregnancy and up to a year postpartum, significantly impact both maternal and infant well-being. Financial hardships, lower quality of life, marital issues, and hazardous behaviors like smoking or suicidal thoughts among women are all associated with perinatal depression [1,2]. In addition, it can impact the development of the fetus or child, resulting in problems including placental anomalies, restricted growth in the womb, or low birth weight. It can also cause pregnancy difficulties like preeclampsia or miscarriages [3,4]. These adverse effects can persist long after the initial postnatal period, manifesting in behavioral challenges, reduced cognitive abilities, difficulty with decision making, and emotional issues later in the child’s life. 

The prevalence rates suggest that up to 20% of women experience significant mental health issues during this period. Early detection and comprehensive care are crucial due to the profound effects these disorders can have [5,6]. Globally, the prevalence of perinatal mood and anxiety disorders (PMADs) has increased significantly, from 18.4 to 40.4 per 1000 deliveries between 2006 and 2015 [7]. This rise is particularly notable in low- and middle-income countries (LMICs), where generalized anxiety disorder affects 22.2% of perinatal women, and 8.3% experience posttraumatic stress disorder [8]. Among urban Black women, the prevalence of PMAD symptoms is alarmingly high at 56%, with substantial associations with pre-pregnancy depression or anxiety and histories of abuse [9].

Organizations like the U.S. Preventive Services Task Force and medical associations recommend screening for mood and anxiety symptoms during pregnancy and postpartum, emphasizing the importance of early intervention [10]. In Europe, the state of perinatal mental health care varies significantly. A recent scoping review covering the WHO European region identified several leading countries in perinatal mental health care, such as Belgium, Finland, Ireland, the Netherlands, Sweden, and the UK. These countries have developed comprehensive PMH policies, screening services, and treatment options, serving as models for others. However, many European countries still lack specific perinatal mental health policies and services, with only 25 out of 53 countries having a specific PMH policy [11]. The UK implements routine screening for perinatal mental health disorders using tools like the Edinburgh Postnatal Depression Scale (EPDS), leading to early identification and timely interventions. In the Netherlands, integrated care involves collaboration among obstetricians, midwives, GPs, and mental health specialists within routine maternity services, enhancing access to care and reducing stigma. Australia mandates comprehensive training on perinatal mental health for healthcare providers, improving their skills in identifying and managing disorders. Sweden allocates specific government funds for perinatal mental health services, supporting specialized care units and research initiatives. Dos for effective policies include universal screening, integrated care, ongoing training, resource allocation, and policy support. Don’ts caution against neglecting cultural sensitivity, underestimating stigma, ignoring the family context, overlooking follow-up care, and failing to evaluate and adapt policies based on evidence. These guidelines aim to improve outcomes for mothers, infants, and families affected by perinatal psychiatric disorders [11].

Untreated mental health conditions during pregnancy can have far-reaching consequences, underscoring the importance of timely diagnosis, appropriate treatment, and support for pregnant women facing mental health challenges. The risks of untreated mental health conditions during pregnancy can impact both the mother and the baby [12] (e.g., increased risk of developing postpartum depression and suicidality [13] and a higher likelihood of engaging in high-risk health behaviors like smoking, substance abuse, and poor nutrition [14]). Moreover, there is an elevated risk of pregnancy complications such as preeclampsia and greater chances of hospital admissions and experiencing mental health relapses [15,16], potentially impacting the mother’s overall well-being and functioning during and after pregnancy. Babies born to women with untreated depression are at risk of prematurity, low birth weight, and intrauterine growth restriction [17,18,19]. Adverse outcomes of untreated maternal depression can affect childhood development, leading to issues like impulsivity, maladaptive social interactions, and cognitive, behavioral, and emotional difficulties in children, with an increased likelihood of delivering small-for-gestational-age newborns. Untreated maternal prenatal depression/anxiety may lead to negative outcomes and increased postpartum effects, impacting the family unit and the child from fetus through to childhood [20,21]. The ramifications of untreated mental health conditions during pregnancy extend beyond the immediate health of the mother [22]. Managing psychiatric issues and pharmacological treatment during pregnancy is complex. However, the benefits of treatment often outweigh the risks [23,24]. These disorders also represent a significant economic burden. In the US, the cost is estimated at USD 14.2 billion per year, while in the UK, it is GDP 8.1 billion per year, primarily due to child morbidity [11]. Investing in early identification and intervention programs not only improves health outcomes but also yields significant cost savings by reducing chronic mental health conditions and associated healthcare utilization.

Advances in research have highlighted the complex interplay of hormonal, genetic, and environmental factors contributing to these disorders, ranging from depression and anxiety to more severe conditions such as postpartum psychosis. Despite growing awareness, significant gaps in research exist (e.g., screening, stigma reduction, and access to specialized care persist), emphasizing the need for concerted efforts to support affected women and their families [5,6]. Healthcare policies play a crucial role in addressing perinatal psychiatric disorders by shaping the availability of mental health services, funding research initiatives, and implementing guidelines for screening and treatment. Policies that prioritize maternal mental health integration within primary care settings and provide adequate training for healthcare providers can help mitigate the underdiagnosis and undertreatment often observed in this vulnerable population. However, it is imperative to fully understand the safety and efficacy of psychotropic therapy.

## 2. Psychotropic Therapy for Pregnant Women

Advancements in psychotropic therapy for pregnant women are crucial in addressing maternal mental health during the perinatal period. Psychotropic medications and psychotherapy have emerged as evidence-based approaches for peripartum depression, underscoring the importance of tailored treatment plans for pregnant women with mental health disorders [25,26]. Despite challenges and the need for further research, advancements in understanding the risks and benefits of psychopharmacological treatment during pregnancy are paving the way for improved care and outcomes for pregnant women facing psychiatric disorders [27].

These psychotropic advancements encompass a diverse range of drugs utilized in the treatment of various conditions. Psychotropic medications function by modulating levels of neurotransmitters, such as dopamine, gamma-aminobutyric acid (GABA), norepinephrine, and serotonin, within the brain [28,29,30,31]. Legal psychotropic medications generally fall into five primary classes: anti-anxiety agents, antidepressants, antipsychotics, mood stabilizers, and stimulants [32]. The most commonly used psychotropic medications during pregnancy are antidepressants, particularly selective serotonin reuptake inhibitors (SSRIs) like citalopram, paroxetine, sertraline, and fluoxetine [33]. Benzodiazepines, such as temazepam, oxazepam, and diazepam, which are classified as anxiolytics and sedatives/hypnotics, are less safe during pregnancy [34]. Antipsychotics include both conventional and atypical antipsychotics, though the consensus is to use them cautiously and at the lowest possible dosage during pregnancy [35]. Mood stabilizers, like lithium, carbamazepine, and lamotrigine, are used to treat bipolar disorder, though carbamazepine and valproate should be avoided, especially in the first trimester, due to the risk of fetal malformations [36]. Infants exposed to psychotropic drugs during pregnancy, especially benzodiazepines and high-dose antidepressants, may experience neonatal toxicity and withdrawal symptoms like jitteriness, crying, and nervousness. While the long-term behavioral and developmental outcomes in children exposed to psychotropic medications during pregnancy are still being investigated, some animal studies have suggested potential effects on brain development and behavior [37]. 

Moreover, the prescription patterns for these psychotropic medications have shifted somewhat over the past two decades, with a greater preference for citalopram, paroxetine, and sertraline among the antidepressants and continued high use of temazepam among the sedative/hypnotics. The overall classes of psychotropic drugs commonly used during pregnancy have remained relatively consistent. However, newer agents have not significantly improved efficacy or speed of action compared to older drugs, and approximately 30% of patients do not respond to current therapies [38,39]. The challenges associated with giving psychotropics to pregnant women include concerns about the risk of birth defects, teratogenicity, harm to the fetus, infant development, and long-term effects on the child. Pregnant women often perceive psychotropic medications to be highly teratogenic, leading to apprehension about their use during pregnancy [40,41]. This apprehension is compounded by uncertainties regarding the effectiveness of these medications in reducing risks associated with mental disorders during pregnancy, such as preventing relapse of major depression or improving outcomes in bipolar disorder and schizophrenia. 

Despite these concerns, it is crucial to carefully weigh the risks and benefits of psychotropic treatments. Ongoing research and more robust evidence are essential to better understand the safety and efficacy of these medications, ensuring that pregnant women receive the best possible care for their mental health needs [42,43]. We need to fully understand if the medication can be adapted by starting at the lowest effective dose and gradually increasing it to minimize risks, as some adverse effects are dose-related, and to avoid suboptimal treatment by the end of titration. Avoiding polytherapy is recommended to reduce the risk of harm, although in some cases, multiple medications may be necessary due to the severity of the disorder [44,45]. Balancing these possibilities and risks is crucial, taking into account the severity of the individual’s psychiatric illness, their response to treatment, and their personal preferences. Advancements in drug development are also necessary to address the gaps in knowledge and improve the safety and efficacy of psychotropic medications for pregnant women. 

Research gaps include a lack of data on the effectiveness of psychotropic medications in mitigating risks associated with mental disorders during pregnancy, the impact of untreated maternal mental illness on pregnancy outcomes, and the long-term effects of fetal exposure to these drugs. More robust evidence is needed in these areas to ensure optimal care for pregnant women [46,47]. It is necessary to understand the optimal dosing strategies, potential drug interactions, and the safety profile of psychotropic medications during pregnancy to ensure the well-being of both the mother and the developing fetus, for each category of these drugs.

### 2.1. Antidepressants

The use of antidepressants during pregnancy and breastfeeding is a complex issue that requires careful consideration of the risks and benefits. Breastfeeding while taking antidepressants can result in the transfer of the medication to the newborn, albeit at significantly lower levels compared to in utero exposure. Breast milk can potentially cause adverse effects in breastfed infants, such as sedation, poor feeding, irritability, and developmental delays [48]. Infants under 6 months old or with immature liver/kidney function are at higher risk, as they have limited capacity to metabolize these drugs. Specific risks have been reported with some medications [49]. To mitigate the risk, the relative infant dose (RID) of a medication, which should be less than 10% of the maternal dose, is used to assess safety during breastfeeding. 

Medications with the most safety data, such as some SSRIs (e.g., sertraline, paroxetine) and mood stabilizers (e.g., valproate, carbamazepine), are generally preferred over higher-risk options [50]. Sertraline is considered well-tolerated in breastfeeding infants, while fluoxetine may be more concentrated in breast milk [51]. There is limited information on the interactions between SSRIs and preterm infants who are breastfed, with only isolated cases of adverse effects reported. Regular monitoring of breastfeeding infants is recommended when mothers are taking antidepressants [52]. Non-pharmacological interventions like psychotherapy should be considered, when possible, to minimize infant exposure. Overall, breastfeeding while on antidepressants is generally considered safe, with minimal transfer of medication to the infant. 

Sertraline is preferred due to its low transfer rate into breast milk, while fluoxetine, though present in higher concentrations in breast milk, is generally well-tolerated. However, caution is advised, especially with preterm infants, as evidence regarding the effects of antidepressants in breast milk on this population is limited. Studies have generally found that SSRIs do not increase the risk of conditions, such as autism, attention-deficit hyperactivity disorder (ADHD), behavioral disorders, developmental speech, language, learning, and coordination disorders, or intellectual disabilities [NO_PRINTED_FORM] [53,54]. However, concerns remain about the risk of spontaneous abortion and the need for further research into long-term effects [55,56]. Moreover, the SSRI dose–response curve tends to be relatively flat, meaning that higher doses do not necessarily lead to significantly greater efficacy. In the context of pregnant women, the flat dose–response curve for SSRIs is particularly relevant due to pharmacokinetic changes during pregnancy. Pregnancy can significantly alter the pharmacokinetics of medications, including SSRIs. Increased blood volume, changes in protein binding, and altered drug metabolism and elimination can all affect SSRI concentrations in pregnant women. Understanding the flat dose–response curves of SSRIs in the general population is essential for guiding dosing decisions in pregnant women. Due to the potential risks associated with both undertreatment and overtreatment of depression during pregnancy, healthcare providers need to consider the unique pharmacokinetic profile of SSRIs in pregnant women. Individualized care that considers factors such as gestational age, maternal health, and potential drug interactions is crucial for optimizing treatment outcomes while minimizing risks. 

Moreover, tricyclic antidepressants (TCAs) have been deemed safe based on clinical observation and data, with no increased risk of malformations reported in infants exposed to them during pregnancy. While they are considered safer than Monoamine Oxidase Inhibitors (MAOIs), they can still pose some risks [57]. TCAs have been associated with low blood sugar and poor tone in newborns, although these symptoms are not thought to cause long-term harm. MAOIs are generally not recommended during pregnancy due to their potential to cause birth defects and other adverse outcomes. However, the specific risks associated with MAOIs are not detailed [58,59]. 

For the treatment of postpartum depression (PPD), brexanolone, a novel antidepressant, has been demonstrated in clinical trials to significantly reduce depression scores in women with moderate to severe PPD. Brexanolone targets the GABA-A receptors, specifically acting as a positive allosteric modulator, which can help regulate emotional behavior and the pharmacological response of GABA receptors [60]. During pregnancy, there are significant changes in neuroactive steroids like allopregnanolone, which play a crucial role in GABA-A receptor function. Allopregnanolone levels increase during pregnancy and decrease rapidly post-delivery, affecting GABA-A receptor sensitivity and potentially contributing to conditions like postpartum depression [61,62]. Brexanolone, along with other GABA-A receptor-selective positive allosteric modulator antidepressants, can offer a therapeutic advantage for mood disorders related to hormonal changes during pregnancy and the postpartum period [63,64]. Brexanalone is approved by the FDA for treating PPD. However, there are limited data on its use in pregnant women, and specific risks remain uncertain. Safety has not been established during pregnancy, and the FDA has not assigned it to a pregnancy category [65]. No data are available on its use in pregnant women to inform drug-related risks for adverse fetal/maternal outcomes, major birth defects, or miscarriage. Some potential risks associated with brexanolone use during pregnancy include preterm delivery, low birth weight, decreased motor tone, etc. [66]. Brexanolone has some limitations, including lack of aqueous solubility, limited accessibility, hospitalization for treatment, lack of an oral product, serious adverse events at high doses [66]. However, the unmet need for synthetic neurosteroids to address PPD outweighs these limitations [67].

In the case of severe psychiatric disorders, Electroconvulsive Therapy (ECT) during pregnancy has been extensively reviewed [68]. While ECT is considered highly effective for severe mental disorders, including depression, bipolar disorder, and schizophrenia, its utilization in pregnancy may be hindered by stigma and access barriers. Physiological changes in pregnancy require special attention during ECT, but serious adverse outcomes are rare. Although complications can occur, they are typically low- or moderate-grade and not life-threatening events. ECT is often used when other treatments have failed or pose risks to the fetus, with particular efficacy in rapidly alleviating critical symptoms like suicidal ideation [69]. Precautionary measures, including maternal and fetal monitoring, are recommended to minimize risks. While there are limitations due to the lack of clinical trials and varying ECT techniques, existing evidence suggests that ECT can be relatively safe and effective during pregnancy, especially when administered with appropriate precautions and a multidisciplinary team approach [58].

### 2.2. Mood Stabilizers

Mood stabilizers like lithium, carbamazepine, and sodium valproate pose teratogenic risks, including congenital malformations, and require careful monitoring during pregnancy. Lithium is highly effective in preventing relapse of bipolar disorder during pregnancy and postpartum. Nonetheless, its use during pregnancy is associated with both benefits and risks due to its association with fetal cardiac malformations [70]. Discontinuation of lithium in pregnant women with bipolar disorder significantly increases the risk of relapse, with approximately 85% experiencing at least one mood episode during pregnancy after discontinuation [71,72]. For instance, a meta-analysis found that lithium was more effective than no lithium in preventing postpartum relapse, with an odds ratio of 0.16 (95% CI: 0.03–0.89) and a number needed to treat of three (95% CI: 1–12). Women with bipolar disorder are at high risk of relapse during the postpartum period. Lithium treatment has been shown to significantly reduce this risk. For example, a study found that women who used lithium during pregnancy had a lower relapse rate compared to those who did not use lithium, with an odds ratio of 0.14 (95% CI: 0.00–6.5) [73]. Lithium exposure during pregnancy has been linked to an increased risk of congenital malformations, particularly cardiac anomalies. A meta-analysis found that lithium exposure during the first trimester was associated with a higher odds ratio of any congenital anomaly (OR: 1.81, 95% CI: 1.35–2.41) and cardiac anomalies (OR: 1.86, 95% CI: 1.16–2.96) [73].

Higher doses of lithium (above 900 mg/day) have been associated with a significantly higher risk of cardiac malformations, with a threefold increase in risk compared to lower doses. Paterno et al. used a large Medicaid database to assess the risk of cardiac defects associated with lithium exposure during the first trimester of pregnancy and found that infants exposed to lithium had an increased relative risk of cardiac defects compared to those exposed to the mood stabilizer lamotrigine and to unexposed infants [74]. Moreover, lithium exposure during the first trimester has also been linked to an increased risk of spontaneous abortion. A meta-analysis found an odds ratio of 3.77 (95% CI: 1.15–12.39) for spontaneous abortion with lithium exposure during the first trimester. While lithium is generally considered safe for the mother, there is a risk of adverse effects on the newborn. Studies have reported an increased risk of neonatal readmission to hospital and other adverse outcomes. During pregnancy, lithium blood levels should be monitored more frequently than usual, preferably weekly in the third trimester [75]. Dosing adjustments may be necessary to stabilize plasma concentrations due to rapid clearance and decreased drug half-life. This helps in maintaining therapeutic levels while minimizing the risk of adverse effects. High-resolution ultrasounds with fetal anomaly scanning at 20 weeks are also recommended to monitor for congenital malformations.

Most clinical guidelines discourage breastfeeding in women treated with lithium due to the potential risks to the infant. The risk of lithium exposure through breast milk is considered significant, and breastfeeding is generally not recommended for women taking lithium. Ideally, pregnancy should be planned during remission from bipolar disorder, and lithium should be prescribed within the lowest therapeutic range throughout pregnancy, particularly during the first trimester and the days immediately preceding delivery. Tapering of lithium during the first trimester could be considered but should be weighed against the risks of relapse. Even the delivery should take place in a specialized hospital where psychiatric and obstetric care for the mother and neonatal evaluation and monitoring of the child can take place immediately after birth. Infants exposed to higher lithium concentrations at birth are at risk for various complications, but discontinuation of lithium before delivery can mitigate these risks [58].

Lamotrigine is another commonly used mood stabilizer during pregnancy, although initial data suggested a possible increased risk of cleft lip/cleft palate [76,77]. However, extensive additional data have not replicated this finding, and increased folic acid intake may help mitigate this risk [78]. Lamotrigine dose adjustments are often needed during pregnancy due to estrogen-induced metabolism changes, with a subsequent rapid decrease in postpartum. While lamotrigine transfers to breast milk, breastfeeding is generally well-tolerated with positive cognitive outcomes in offspring, although rare cases of infant respiratory difficulties due to maternal lamotrigine toxicity have been reported [79]. Carbamazepine therapy during pregnancy likely does not increase the risk of congenital malformations [80]. On the other hand, valproic acid is contraindicated for use in pregnant women due to its association with a range of adverse outcomes in offspring, including major congenital malformations, behavioral issues, autism, and developmental delays [80]. While valproate and carbamazepine are commonly used as mood stabilizers, the evidence supporting their efficacy in this role is limited. Studies suggest that valproate is not superior to other options like lithium or atypical antipsychotics [81].

Valproate is associated with teratogenic effects, making it unsuitable for pregnant women. It significantly increases the risk of serious birth defects, including neural tube defects like spina bifida [82]. Nonetheless, in some cases, the benefits of managing bipolar disorder may outweigh the risks, but this decision should be made carefully with a healthcare provider [83]. Carbamazepine has reasonable evidence supporting its antimanic effect, but it is not the first-line choice [84]. Lithium, valproate, or atypical antipsychotics are often preferred. Like valproate, carbamazepine carries teratogenic risks during pregnancy, and in specific situations, it may be necessary to continue carbamazepine treatment to prevent relapses, but close monitoring is crucial [85].

The ethical dimension of benefit–risk management is essential. Pregnant patients need reassurance, as stress from worrisome information can lead to relapses. Clinicians must weigh maternal relapse risk against antenatal exposure to mood stabilizers. Individualized decisions are crucial, considering each patient’s unique circumstances. Due to the significant risks associated with the use of valproate and carbamazepine during pregnancy, regulatory bodies have taken steps to restrict their use in this population. For instance, the U.S. Food and Drug Administration (FDA) has issued warnings about the use of valproate (valproic acid) and carbamazepine during pregnancy [86]. They emphasize the potential for birth defects and other adverse outcomes. Healthcare providers are advised to carefully weigh the risks and benefits when considering these medications for pregnant patients. 

Although lithium is not entirely risk-free, it is generally considered safer than valproate and carbamazepine. Lithium exposure may slightly increase the risk of cardiac malformations in the fetus, but it effectively prevents relapses in bipolar disorder. However, this is confounded by the inability to control for other potentially teratogenic factors [87,88]. Therefore, regular blood level monitoring is crucial to maintain therapeutic levels while minimizing risks. Moreover, atypical antipsychotics like quetiapine, olanzapine, and lurasidone are commonly used during pregnancy; even though some risks exist (e.g., gestational diabetes), they are generally considered safer than older mood stabilizers. These drugs help manage acute episodes and prevent relapses [89]. Cognitive-behavioral therapy (CBT) and interpersonal therapy can complement medication [90]. 

Nonetheless, research on mood stabilizers in pregnant women should evolve to address critical gaps and improve clinical decision making, especially to access long-term child development outcomes and individualized risk–benefit assessment.

### 2.3. Antipsychotics

Antipsychotic medications, both conventional and atypical, generally do not pose an increased teratogenic risk, although some complications may arise in the infant’s immediate postnatal period [35,91]. During pregnancy, antipsychotics are prescribed for approved conditions like schizophrenia, bipolar disorder, and depression but are also commonly used off-label for sleep and anxiety issues. A study using Medicaid data found that during the first trimester, antipsychotic use did not significantly raise the risk of birth defects after adjusting for other factors [92]. However, risperidone showed a small increase in overall malformations and a potential risk for cardiac malformations, indicating a potential safety concern for its use during this period [93]. This risk might extend to paliperidone, risperidone’s primary active metabolite. Some atypical antipsychotics, like olanzapine and quetiapine, have also been linked to higher rates of gestational diabetes due to their metabolic effects [58]. There is insufficient evidence to determine which antipsychotic is the safest option for use during pregnancy. The use of anticholinergic drugs alongside antipsychotics should be minimized due to potential teratogenicity. If necessary, they should be used at the lowest effective dosage [94,95].

Research indicates that a significant proportion of pregnant women, particularly those with bipolar disorder, require polypharmacy due to multiple comorbidities, with the number of medications ranging widely from 3 to 13.6 during pregnancy [96]. Polypharmacy, particularly with second-generation antipsychotics and other psychotropic medications, is linked to higher risks of neonatal morbidity, including prematurity, NICU admission, and congenital malformations [97]. Pregnant adults managing ADHD often explore non-drug strategies, but severe cases may necessitate stimulant treatment during pregnancy to improve symptom control. However, there is limited information on the safety of methylphenidate during pregnancy. Most data come from animal studies, and human studies are scarce. Animal studies suggest potential teratogenic effects, including skeletal variations and increased risk of spina bifida [98]. Methylphenidate can cross the placenta, potentially affecting the developing fetus. It is established that the risk of cardiac defects is statistically nonsignificant, but there is a significant association with gastroschisis (a congenital abdominal wall defect) [99,100]. Pooled data from Nordic Health Registries confirmed a small rise in cardiac malformations specifically associated with methylphenidate, whereas no heightened risk was observed with amphetamine-based stimulant use in the first trimester [58].

Moreover, women often use benzodiazepines to manage anxiety, either as needed or regularly. Like lamotrigine, initial studies raised concerns about the risk of cleft lip and cleft palate, but larger reviews have debunked this link [101]. A comprehensive European study found no heightened congenital malformation risk with first-trimester benzodiazepine use. Benzodiazepines, commonly used as anxiolytics, carry a risk of withdrawal symptoms in infants and may lead to neonatal toxicity if used during pregnancy. High doses and long-term use should be avoided, and alternative treatments should be considered if sedation is necessary. However, these medications have been linked to neonatal adaptation signs, persisting for weeks postpartum [102]. Lorazepam is preferred for breastfeeding due to its shorter half-life [103]. Insomniatrazodone is often prescribed at low doses as a safe hypnotic. However, is not usually recommended during pregnancy. Trazodone falls into FDA pregnancy category C, indicating that its risk during pregnancy is not well established [104]. Zolpidem, another option, has not shown increased malformation risk and has minimal impact on breastfeeding [56,103,105].

**Figure 1:**
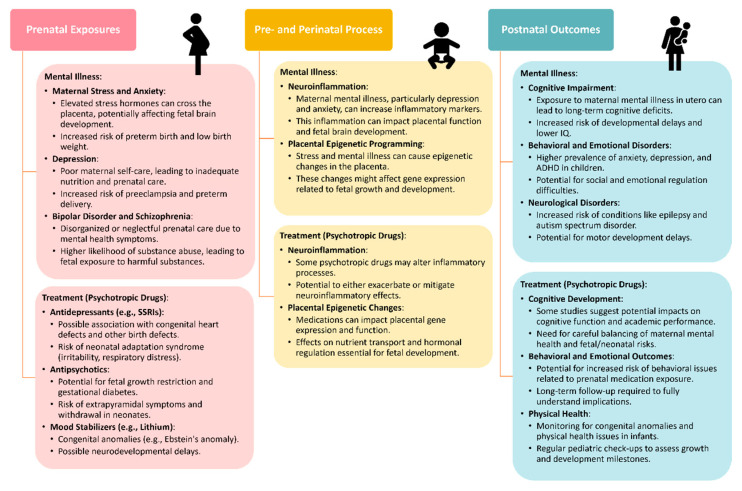
Adverse outcomes caused by mental illness or its treatment across different stages. These include prenatal exposure [106], pre- and perinatal process [107], postnatal outcomes [108].

During the initial assessment, clinicians should discuss treatment preferences with women and consider psychotherapy referrals as appropriate. Many women with chronic mental illness require maintenance medications, and the choice of psychotropics during pregnancy should prioritize the one most effective for the individual patient. Careful discussion and documentation of medication use during pregnancy are essential for informed decision making and legal purposes. Standardized measures completed monthly across pregnancy, such as the Patient Health Questionnaire-9 or EPDS, can help monitor symptoms and guide treatment adjustments. The Mood Disorders Questionnaire is useful for screening bipolar disorder. Dosing changes during pregnancy and postpartum should be managed carefully, with adjustments made based on symptom control and metabolic changes. Overall, adequate evaluation and treatment of psychiatric disorders during pregnancy are crucial for optimizing maternal and infant outcomes. While some evidence suggests potential benefits of antidepressant use during pregnancy, ongoing research is needed to better understand the risks and benefits of pharmacotherapy in this population (Figure 1).

### 2.4. Adverse Outcomes and Risks of Psychotropic Medication Use

Pregnant patients navigating mental health challenges may necessitate a variety of psychotropic medications tailored to their individual needs. Antipsychotic use during pregnancy poses potential risks, including increased chances of gestational diabetes mellitus, low birth weight, cesarean delivery, and prematurity [35]. Exposure to these drugs during pregnancy has also been linked to congenital abnormalities, preterm birth, and metabolic disturbances that could affect fetal growth [109]. Second-generation antipsychotics, in particular, may heighten the risk of diabetes mellitus and weight gain in pregnant individuals [89]. However, the overall impact on neonatal outcomes remains complex due to confounding variables and the lack of high-quality evidence from randomized controlled trials in pregnant women.

Failure to receive appropriate treatment or discontinuing treatment during pregnancy can increase the risk of maternal relapse, hospital admissions, and overall impact on the mother’s well-being and functioning. Antipsychotic medication is used to manage conditions, such as bipolar disorder, schizophrenia, depression, anxiety, and insomnia. Without treatment, patients may experience relapse rates as high as 71%, particularly with bipolar disorder. Managing psychiatric issues during pregnancy is complex, considering both biological and personal factors. Hormonal changes, emotional stress, and lifestyle adjustments can increase the risk of mental health problems, including the exacerbation of existing conditions such as depression and anxiety disorders. Psychosis that arises during pregnancy can result in poor cooperation and care during delivery, posing risks to both the mother and the fetus [110,111]. Untreated maternal psychiatric disorders may impair cognitive function and brain development in the fetus and elevate the risks of self-harm, substance abuse, and inadequate prenatal care. The potential to cause birth defects or developmental abnormalities in the fetus is a critical factor in the decision-making process when prescribing antipsychotic drugs during pregnancy [111,112]. Antipsychotic medications can easily cross the placenta, exposing the fetus to potential harm [113]. Balancing the benefits of treating the mother’s psychiatric condition with the potential risks to the fetus poses a significant challenge for clinicians [114].

Evidence suggests possible side effects and risks associated with psychotropic medication, emphasizing the need for a thorough evaluation of both anatomical malformations and long-term behavioral effects. For example, SSRIs have shown some protective effects against late preterm birth, very preterm birth, and cesarean section. Nonetheless, neonates exposed to SSRIs in utero may exhibit symptoms of Neonatal Abstinence Syndrome (NAS) and Delayed Neonatal Adaptation, including respiratory, gastrointestinal, neuromuscular, and central nervous system issues [115]. NAS is when a newborn experiences withdrawal symptoms after being exposed to certain drugs, most often opioids, in the womb before birth. When a pregnant woman takes opioid drugs or certain other addictive substances, these substances can pass through the placenta and cause the baby to become dependent on them. After birth, when the baby is no longer receiving the drug, they can experience withdrawal symptoms, such as irritability, excessive crying, tremors, seizures, poor feeding, diarrhea, and others. NAS symptoms typically appear within 1–3 days after birth but can sometimes take up to a week to manifest. Treatment often involves medications to help wean the baby off the substance and supportive care [116]. The Delayed Neonatal Adaptation refers to a delay in the normal physiological changes that occur in a newborn after birth. This can happen when a newborn is exposed to certain medications or substances in utero, such as antidepressants, benzodiazepines, or opioids [117]. Unlike NAS, which involves withdrawal symptoms, Delayed Neonatal Adaptation is characterized by a slower transition to extrauterine life, with symptoms like respiratory distress, hypoglycemia, temperature instability, and feeding difficulties. These symptoms may not appear until several days after birth, unlike the more immediate withdrawal symptoms seen in NAS. Treatment focuses on supportive care and monitoring the newborn’s adaptation to life outside the womb. The key difference is that NAS involves active withdrawal from substances, while Delayed Neonatal Adaptation refers to a slower, more gradual adjustment to life outside the womb after in utero exposure to certain medications or drugs. Both conditions can have serious consequences for the newborn and require specialized medical care. The incidence of NAS varies widely, with some variability based on the specific SSRI used [118]. The overall incidence of NAS in the United States increased almost 300% during 1999–2013, from 1.5 to 6.0 cases per 1000 hospital births. In 2016, the overall incidence rate of NAS was 6.7 per 1000 in-hospital births. Neonatal Abstinence Syndrome incidence rates increased from 1.5 to 8.0 per 1000 hospital births in the United States from 2004 to 2014. The incidence rate of NAS in Pennsylvania remained highest in the state’s northwestern region, increasing from 23.7 NAS cases per 1000 live births in 2019 to 30.8 NAS cases per 1000 live births in 2020. In 2013, NAS incidence ranged from 0.7 cases per 1,000 hospital births (Hawaii) to 33.4 cases per 1000 hospital births (West Virginia) across 28 states. Overall, the incidence of drug-exposed newborns is reportedly 3–50%, depending on the specific patient population, with urban centers usually reporting higher rates. Developmental outcomes in infants exposed to SSRIs in utero have been compared to those exposed to maternal depression without antidepressant treatment and those not exposed to either [119]. While no significant differences were found in cognitive development, infants exposed to SSRIs showed worse psychomotor development scores at certain ages. However, these differences disappeared by 78 weeks of age [120,121,122].

A recent study by Bruno et al. analyzed data from pregnant women with psychiatric disorders and their children from Denmark, Finland, Iceland, Norway, and Sweden. They found that prenatal exposure to antipsychotics did not significantly increase the risk of neurodevelopmental disorders or poor academic performance in children. Among over 213,000 children studied, researchers found that exposure to antipsychotic medication during pregnancy did not lead to a significantly higher risk of neurodevelopmental disorders such as intellectual developmental disorders, speech or language disorders, or learning disorders. The study examined the academic performance of children exposed to antipsychotics prenatally and found no substantial increase in poor performance in mathematics or language arts based on standardized national school tests [123]. Consistent results across various individual antipsychotic medications and different trimesters of exposure were observed, suggesting that the lack of increased risk held true regardless of the specific medication or timing of exposure during pregnancy. Overall, the study provides reassurance to clinicians and women managing mental illness during pregnancy that the use of antipsychotic medication is not significantly associated with adverse neurodevelopmental outcomes or learning difficulties in children. The study’s large multinational cohort and comprehensive analysis underscore the importance of population-based cohort studies in providing robust real-world evidence on the long-term safety of medications during pregnancy [22].

Galbally et al. noted that the associated risks for severe mental disorders or their treatments on pregnancy and infant outcomes should consider the prescribing practices, including the likelihood of exposure to polypharmacy and a range of potential confounding co-morbidities and exposures. The study examined real-world prescribing practices for pregnant women with severe mental illness across two hospitals in Australia. It included 535 women with various severe mental illnesses, such as psychotic disorders and bipolar disorders [70]. The majority of these women were prescribed psychotropic medication during pregnancy, with a significant portion receiving more than one class of medication. Variations in prescribing practices were observed between hospitals and across different mental disorders. The study also highlights elevated rates of pregnancy complications and co-morbidities among women with severe mental illness compared to the general population. Differences in medication use and management strategies suggest the need for comprehensive guidelines tailored to managing severe mental illness in pregnancy. Additionally, the study underscores the importance of considering factors like polypharmacy and co-morbidities in research on pregnancy outcomes associated with SMI and its treatments [25].

Peripartum depression affects approximately one in seven women, with many experiencing episodes before or during pregnancy. Discontinuation of antidepressants during pregnancy increases the risk of relapse, with rapid relapses occurring in the first trimester [124,125]. Nonpharmacologic treatments like psychotherapy are viable options, often combined with antidepressants for moderate-to-severe depression or used alone for mild cases [126]. Medication use during pregnancy is common, with a significant percentage of women taking antidepressants. Preterm birth rates are elevated in women with depression, whether medicated or not, and the association between antidepressant use and preterm birth is complex, depending on the specific medication and the successful treatment of depression. NAS can occur in infants exposed to antidepressants in utero, with symptoms ranging from neuromuscular to respiratory difficulties. However, the mechanism remains unclear, with hypotheses including withdrawal, serotonin toxicity, and teratogenic effects. Developmental outcomes of children exposed to antidepressants in utero are varied, with some studies showing lower psychomotor scores initially but no significant differences in cognitive development or intelligence later on [127,128]. Pharmacokinetic changes during pregnancy affect antidepressant metabolism, necessitating dose adjustments to maintain therapeutic levels. Genetic polymorphisms can also influence drug metabolism, highlighting the need for personalized treatment approaches [129]. Ongoing studies like OPTI-MOM and FANSMAT aim to provide further insights into optimal medication management and fetal implications [58,130,131]. Therefore, the decision to prescribe antipsychotic medications during pregnancy thus involves careful balancing of potential benefits against these known risks, emphasizing the need for personalized treatment strategies guided by the latest research and clinical evidence.

## 3. Individualized Dosing and Therapeutic Monitoring

### 3.1. The Need for Personalized Treatment in Pregnancy

Pregnancy induces significant changes in the body’s pharmacokinetics and pharmacodynamics, impacting the way psychotropic medications are processed and interact within the body. These changes include alterations in drug absorption, distribution, metabolism, and elimination. During pregnancy, physiological changes, such as delayed gastric emptying, increased plasma volume, altered protein binding, and enhanced hepatic metabolism, can affect the levels and effectiveness of psychotropic drugs. Additionally, the increased activity of metabolic enzymes like cytochrome P450 and changes in renal blood flow can lead to variations in drug elimination rates. These modifications can result in lower drug levels, potentially affecting the efficacy and safety of psychotropic medications during pregnancy [132]. It is crucial for healthcare providers to consider these alterations in pharmacokinetics and pharmacodynamics when prescribing psychotropic medications to pregnant women, emphasizing the need for cautious dosing, regular monitoring, and a thorough risk–benefit assessment to ensure the well-being of both the mother and the developing fetus [133].

Therefore, it is highly important to understand pharmacokinetics for dosing regimens. Proper dosing ensures therapeutic efficacy for the mother while minimizing fetal exposure. Overdosing due to altered pharmacokinetics can lead to toxicity [134,135]. Close monitoring and dose adjustments are critical, as each woman’s pharmacokinetic profile varies. Individualized dosing accounts for these differences. Moreover, the timing of drug administration plays a crucial role in determining peak plasma concentrations in the mother [136]. Administering a drug before meals, particularly on an empty stomach, can result in higher peak levels. Additionally, timing also impacts trough concentrations, which are the lowest levels of the drug in the bloodstream. Consistent dosing intervals are essential for maintaining therapeutic levels. The drug’s half-life, or the time it takes for half of the drug to be eliminated from the body, influences how often it needs to be administered [137]. Drugs with longer half-lives may require less frequent dosing. In the fetal compartment, drugs can cross the placenta, affecting fetal exposure. The timing of drug administration relative to delivery can influence fetal levels, with administration close to delivery potentially resulting in higher fetal drug levels. Fetal organs develop at varying rates, and exposure during critical periods can impact organogenesis. Some drugs may also cause neonatal withdrawal or affect neonatal behavior [138].

Balancing therapeutic efficacy and fetal safety requires careful consideration of various factors. The optimal timing of drug administration should be based on the drug’s pharmacokinetics to maintain consistent therapeutic levels. A risk–benefit assessment is crucial, considering both maternal health benefits and potential fetal risks such as teratogenicity, neonatal toxicity, and long-term effects. An individualized approach is essential, considering the severity of the maternal condition, alternative treatment strategies, and collaborative decision making involving obstetricians, psychiatrists, and pharmacists. Adjusting timing or considering alternative medications may be necessary in some cases to optimize outcomes for both the mother and fetus.

### 3.2. Therapeutic Drug Monitoring in Pregnant Women

TDM is crucial during pregnancy due to physiological changes that can alter drug pharmacokinetics, potentially affecting treatment efficacy and toxicity for both mother and fetus. TDM aims to optimize drug dosing regimens, enhance treatment effectiveness, and minimize toxicity (Table 1) [139]. This approach is particularly valuable for medications with narrow therapeutic indices and significant inter-individual variability. Despite these benefits, the clinical impact and cost-effectiveness of TDM during pregnancy remain uncertain, largely due to challenges in conducting clinical pharmacokinetic studies in pregnant women. However, regulatory agencies are encouraging the inclusion of pregnant women in clinical trials to improve pharmacotherapy understanding in this population, highlighting the utility of TDM in guiding safer and more effective treatment strategies [140]. TDM has been proposed for various medications during pregnancy, including antiepileptics, antidepressants, and antibiotics. Its utility lies in its ability to tailor drug dosages to the unique physiological changes that occur during pregnancy, ensuring that therapeutic levels are maintained while minimizing adverse effects. This is particularly critical when conventional measures, such as coagulation indices or glycemia, are inadequate for assessing therapeutic effects or toxicity. TDM’s role is well-established in epilepsy treatment, where it helps maintain seizure control by adjusting drug dosages based on plasma levels [141,142].

Recent advancements in nanotechnology and point-of-care (PoC) monitoring have significantly enhanced the utility of TDM. Innovations such as smartphone-integrated platforms have revolutionized real-time monitoring, making personalized therapy more accessible and accurate for pregnant women [127]. These technologies enable non-invasive sampling and miniaturization of analytical devices, facilitating more convenient and cost-effective TDM. This is especially beneficial for vulnerable populations, including neonates and children, where traditional monitoring methods may be challenging [143,144]. The practical utility of TDM is further demonstrated in the context of antiepileptic drug monitoring during pregnancy. Research has shown that dosage adjustments are necessary to maintain therapeutic levels due to altered drug disposition starting in early pregnancy [145,146,147,148].

Although these adjustments may not always improve overall seizure control, they underscore the importance of regular monitoring and individualized therapy [149]. Wearable biosensors incorporating nucleic-acid-based assays represent a significant advancement in health monitoring technology. These sensors offer improved stability, analytical performance, and clinical applicability, making TDM more effective and user-friendly [150]. However, the success of nucleic-acid-based point-of-care diagnostics has led to a paradigm shift, offering improved stability, analytical performance, and clinical applicability. This integration opens new avenues for non-invasive, real-time monitoring of health and personalized medicine, paving the way for enhanced capabilities in wearable health monitoring technologies [143,151,152]. In conclusion, TDM’s utility in pregnancy cannot be overstated. It provides a critical tool for optimizing drug therapy, ensuring both efficacy and safety. Advances in portable and wearable monitoring technologies promise to further enhance the accessibility and accuracy of TDM, making it an indispensable component of personalized medicine for pregnant women. Continued research and development in this area are essential to fully realize the potential of TDM in improving maternal and fetal health outcomes [140,153].

#### 3.2.1. Sampling and Methods for Drug Level Measurement

Traditional blood sampling is a common method for TDM, allowing for direct measurement of drug concentrations in the bloodstream. Regular blood tests help ensure that therapeutic levels are maintained and can guide dose adjustments if needed. However, repeated blood draws can be inconvenient and burdensome for pregnant women. Dried Blood Spots (DBSs) involve collecting small blood samples on filter paper, which can then be analyzed for drug concentrations. This method is less invasive compared to traditional blood draws and can be done at home, providing a convenient option for monitoring drug levels during pregnancy. DBS is particularly useful for drugs that have a narrow therapeutic index and require close monitoring. DBS sampling has been used for over 50 years, particularly for newborn screening programs to detect inborn errors of metabolism. In recent years, the use of DBS sampling has accelerated in epidemiological research and clinical applications for measuring a wide range of analytes, including environmental exposures, trace elements, and drugs.

Saliva samples can be used to measure drug levels, offering a non-invasive alternative to blood tests. This method is particularly useful for drugs that are present in saliva in concentrations that correlate well with blood levels. Saliva monitoring can be carried out at home and is generally well-tolerated by pregnant women. The same goes for urine tests. Samples are used to monitor drug metabolites, providing information on drug metabolism and excretion. This method is useful for assessing compliance and detecting potential drug interactions. Urine tests are non-invasive and can be easily obtained from pregnant women.

The need for non-invasive methods is particularly important in pregnant women, as repeated blood draws can be challenging and may cause discomfort or anxiety. Non-invasive methods like DBS, saliva monitoring, and urine tests are preferable as they minimize the burden on the patient and can be easily integrated into routine prenatal care. However, it is important to note that not all drugs have well-established correlations between blood, saliva, or urine levels, and the choice of monitoring method should be based on the specific drug and its pharmacokinetic properties. Additionally, the interpretation of drug levels may be complicated by the physiological changes that occur during pregnancy, such as increased blood volume and altered drug metabolism.

As a suggestion for further development in this field, AI-photonic technology could potentially be used to analyze DBS samples. Combining artificial intelligence with photonics could enhance the sensitivity and accuracy of drug-level detection. AI-photonic systems could rapidly process and analyze DBS samples with high precision, making them ideal for real-time monitoring of drug concentrations. Integrating AI algorithms would allow these systems to learn and adapt to various drug profiles, potentially improving the reliability of the data obtained [154,155]. This innovative approach could facilitate more efficient and non-invasive drug monitoring, supporting personalized treatment plans by providing timely and accurate information about drug levels, even amidst the physiological changes of pregnancy. Further research and development in this area could pave the way for significant advancements in TDM for pregnant women.

#### 3.2.2. Wearable Sensors for Pregnancy

Wearable sensors offer promising potential for monitoring the health of both mothers and their babies throughout pregnancy. It is a critical area because too many women still face risks during pregnancy and childbirth. The integration of wearable sensors in monitoring the health of pregnant women and their babies represents a critical advancement within the context of psychotropic treatment during pregnancy [156,157]. Wearable sensors to monitor stress in pregnant women present several key points for consideration, such as sensor type, placement, battery life, and data collection. In the literature, sensor types like electrocardiogram (ECG), photoplethysmography (PPG), and galvanic skin response (GSR) are recognized as reliable for stress monitoring [158]. Proper implementation of these sensors can significantly improve maternal health outcomes by reducing stress-related fatalities. Reducing stress can lower the risk of complications such as preterm birth and low birth weight. Alim et al.’s systematic review underscores the importance of monitoring maternal health due to the high number of daily deaths from pregnancy and childbirth complications [159]. The review covers various sensors, including those for monitoring fetal ECG (FECG), fetal heart rate (FHR), fetal movement (FM), and maternal physical activities. ECG, PPG, and GSR sensors can continuously monitor physiological indicators of stress, with machine learning algorithms analyzing data to classify stress levels, allowing for timely interventions. Wearable sensors can also provide continuous monitoring of vital signs and physical activity, offering insights into the mental health status of pregnant women. Early detection of abnormal patterns can prompt timely psychological or psychiatric interventions. However, challenges such as sensor placement, comfort, and algorithmic accuracy must be addressed to achieve optimal performance. Enhanced prenatal care can be achieved through wearable devices enabling remote monitoring, reducing the need for frequent in-person visits, which is beneficial for those with psychological conditions that make travel or frequent appointments challenging. Real-time data can be shared with healthcare providers, allowing for immediate adjustments to care plans. Data from wearables can be used to tailor interventions specific to the individual’s needs, improving outcomes for both the mother and the fetus. Monitoring physical activity and other behaviors can provide insights into the lifestyle factors affecting mental health, leading to more effective behavioral interventions.

Strategic placement of these sensors and the design of comfortable wearables are essential to ensure accurate data collection. Wearables need to be designed with the comfort of pregnant women in mind to ensure compliance and accurate data collection. Developing robust algorithms that can accurately interpret the data from wearable sensors is essential, including improving noise and artifact removal techniques to ensure the reliability of the data. Integrating machine learning algorithms enhances the analysis and classification of stress levels [158]. More studies are needed to test these sensors in real-life conditions, as most current research has been conducted in controlled environments. This will help in understanding how these devices perform during everyday activities and over extended periods. Wearable sensors can significantly enhance the health outcomes of pregnant women, particularly in managing psychological and psychiatric conditions. By reducing stigma associated with mental health issues during pregnancy, these sensors normalize the monitoring and management of maternal mental health. This normalization encourages women to seek timely assistance and engage in open discussions about treatment options with healthcare providers.

Li et al. explored user perceptions of mHealth technologies for health and well-being monitoring in pregnancy, highlighting both pregnant women and healthcare providers’ openness to such technologies for better monitoring and support, despite concerns about usability and additional workload for healthcare providers [160]. The integration of smartphone platforms and home-based monitoring systems enhances data accuracy and convenience, facilitating timely adjustments to treatment plans based on objective metrics. This proactive approach supports personalized care and contributes to evidence-based guidelines for psychotropic use during pregnancy. Smartphone apps and attachments enable real-time monitoring, allowing patients to track drug levels and receive alerts when adjustments are necessary. This integration enhances patient empowerment and reduces the need for frequent hospital visits. Real-time monitoring enables healthcare providers to continuously track drug levels, maternal health, and fetal well-being, allowing for prompt adjustments based on changing conditions [140,153]

### 3.3. Methods for Individualized Dosing

#### 3.3.1. Population-Based and Physiologically Based Pharmacokinetic Modelling and Simulation

Personalized dosing strategies based on pharmacokinetic modeling are transforming the landscape of drug therapy for pregnant women, providing tailored approaches to optimize maternal health and fetal safety. This process begins with the development of population-based (popPK) and physiologically based pharmacokinetic (PBPK) models. By gathering pharmacokinetic data, such as plasma concentrations, from pregnant individuals and employing statistical modeling techniques, factors like age, weight, gestational age, genetic variations, trimester-specific alterations in the activity of drug-metabolizing enzymes like CYP1A2, CYP2D6, and CYP3A4, and other relevant variables are considered [161]. The goal is to anticipate drug exposure in diverse patient groups and inform dosing recommendations accordingly.

Once these models are established, personalized dosing strategies can be put into practice. This individualized approach utilizes patient-specific data, such as renal function and liver enzymes, to fine-tune dosing regimens. Clinicians can then adjust the dose and frequency of medication based on the patient’s pharmacokinetic profile, thereby ensuring optimal therapeutic outcomes while minimizing risks [44]. Implementing personalized dosing strategies significantly enhances maternal and fetal safety through optimized dosing. Clinicians can make evidence-based decisions by leveraging pharmacokinetic data to guide treatment choices, achieving a delicate balance between maternal mental health and fetal well-being [162]. Regulatory agencies also play a crucial role in considering pharmacokinetic data when evaluating drug safety during pregnancy, ultimately leading to informed labeling recommendations. By tailoring drug therapy to individual patient characteristics, clinicians can optimize treatment outcomes and enhance overall maternal and fetal health [163].

However, the gaps in knowledge regarding the pharmacokinetics of certain antidepressants in pregnant women are still persistent. Data on the pharmacokinetics of antidepressants are scarce and not widely available. There is limited reporting of PK parameters and insufficient sampling (e.g., some studies only collect samples at delivery, limiting the understanding of how drug concentrations change over the course of pregnancy), as well as small sample sizes [164]. In Yue et al.’s review, they report findings from 40 publications on 15 antidepressants studied in 961 pregnant women. They found PK data for multiple drugs: desvenlafaxine, milnacipran, and viloxazine. Only six studies reported PK parameters such as area under the concentration–time curve (AUC), clearance (CL), or volume of distribution (V). Four studies collected samples at multiple time points following a single dose, while ten studies reported information at delivery only. Eight studies were case reports with only one patient for each drug. They found limited PK data, mostly from small studies with poor quality and incomplete reporting. Most data were collected at delivery, but longitudinal data with maternal trough concentrations were often missing [164]. Despite challenges, some findings suggest decreased drug exposure during pregnancy, requiring increased dosing. For example, George et al. developed a PBPK model to predict sertraline dosing in pregnancy, considering the physiological changes that occur during gestation. They constructed a model using data from the literature, including sertraline properties, in vitro metabolism studies, and physiological changes in pregnancy. The PBPK model accurately predicted sertraline pharmacokinetics in both nonpregnant and pregnant populations. They found that physiological changes in pregnancy led to increased clearance of sertraline, potentially resulting in under-dosing if nonpregnancy doses were used. The average fold error (AFE) number was used to calculate the accuracy for Cmax and AUC24 in the second and third trimesters. In the second trimester, the AFE value for Cmax was 0.6, and in the third, it was 0.5. In the second trimester, the AFE value for AUC24 was 0.9, and in the third, it was 0.7. A range of 1.5–1.7 was the precision, as indicated by the average absolute fold error (AAFE) value for both Cmax and AUC24. Regarding Cmax, four out of six participants and six out of eight subjects, respectively, were within a twofold error of the observed values during the second and third trimesters. To facilitate clinical application, they converted the PBPK model into a web-based dosing tool. The study highlights the utility of PBPK modeling in optimizing drug dosing during pregnancy and suggests its potential for broader application in predicting drug exposure across gestational ages [165].

Specific examples for each psychotropic drug can also be given. Precision dosing of venlafaxine during pregnancy can be accessed using pharmacokinetic modeling aimed to assess changes in maternal and fetal venlafaxine levels throughout pregnancy and identify appropriate doses to maintain therapeutic levels. Venlafaxine plasma concentrations decrease during pregnancy due to changes in maternal physiology and CYP2D6 polymorphisms. A significant portion of pregnant women, especially those with extensive metabolizer (EM) and ultra-rapid metabolizer (UM) phenotypes, have venlafaxine plasma concentrations below therapeutic levels (<25 ng/mL), which increases as pregnancy progresses. Daily dose adjustments are necessary for all phenotypes throughout pregnancy. For EM phenotypes, doses of 225 mg in the first trimester, 262.5 mg in the second trimester, and 375 mg in the third trimester are suggested. For UM phenotypes, a daily dose of 375 mg throughout gestation is recommended. For PM phenotypes, a dose of 37.5–112.5 mg daily throughout pregnancy is suggested [166].

Regarding mood stabilizers, lamotrigine has been increasingly explored in pregnancy. Polepally et al. investigated changes in lamotrigine clearance during pregnancy using a model-based approach. They collected data from pregnant women receiving lamotrigine therapy and analyzed them using a population-based, nonlinear, mixed-effects model. The results showed that most women experienced a substantial increase in lamotrigine clearance by the end of pregnancy, while a minority had a minimal increase [167]. This increase in clearance could lead to lower lamotrigine blood concentrations, necessitating more frequent dosage adjustments and closer monitoring in some pregnant women with epilepsy. Additionally, postpartum doses should be tapered to preconception levels within three weeks of delivery to avoid potential adverse effects [168]. Berezowska et al. focused on PBPK modeling of lamotrigine and raltegravir during pregnancy. Using data from a meta-analysis, they developed pregnancy-specific models for both drugs and validated them against clinical data. The models successfully predicted changes in drug exposure during pregnancy, accounting for physiological changes and enzyme activity variations. This approach provides a valuable tool for predicting drug exposure and optimizing dosing regimens for pregnant women, especially in the absence of extensive clinical data [169]. Silva et al. aimed to replicate and reassess PBPK models for betamethasone and buprenorphine in pregnant women. They verified the ability of the models to replicate previous findings and assessed their performance using additional clinical data. The betamethasone model was successfully replicated and showed good agreement with observed data, while the buprenorphine model was not reproducible. This emphasizes the importance of model reproducibility and validation for ensuring the reliability of PBPK modeling in pregnancy [170].

The safety of antipsychotic use during pregnancy is a complex issue, and identifying the safest options can be challenging due to ethical considerations and limited clinical trial data involving pregnant women. Zheng et al. developed a PBPK model for olanzapine in pregnant women to predict changes in drug exposure during pregnancy. The model was validated using pharmacokinetic data from non-pregnant populations and then extrapolated to predict drug levels throughout pregnancy. The findings suggest that under a 10 mg daily dose, olanzapine exposure in pregnant women only experiences minor changes (less than 28%) throughout pregnancy. The reduction in cytochrome P4501A2 (CYP1A2) activity during pregnancy seems to be counteracted by the induction of other enzymes, particularly glucuronyltransferase1A4 (UGT1A4). The study concludes that dose adjustment of olanzapine during pregnancy may not be necessary if effective treatment is established before pregnancy and fetal toxicity concerns are addressed. However, the study acknowledges the need for further research on fetal exposure to olanzapine and emphasizes the importance of individualized dosing and TDM during pregnancy [171].

Another study aimed to investigate the impact of pregnancy on the pharmacokinetics of risperidone and its active metabolite paliperidone by analyzing their serum concentrations in a pregnant woman and her newborn. A PBPK model was constructed for both drugs in adults, pediatric, and pregnant populations using the Simcyp simulator. These models were then applied to the pregnant woman and her newborn, generating “virtual twins” to predict drug pharmacokinetics. Risperidone was not detected in the serum of the pregnant woman or her newborn, while paliperidone concentrations were within a specific range. The PBPK models accurately predicted paliperidone pharmacokinetics across populations. The study also examined the effects of pregnancy on drug metabolism and renal function, as well as evaluating various methods for estimating neonatal renal function to optimize drug dosing in pediatric populations. The study provides insights into individualized drug dosing in pregnant and pediatric patients using PBPK modeling, potentially enhancing precision dosing strategies in these populations [172].

Leutritz et al. investigated alterations in blood concentrations of psychotropic drugs during pregnancy and lactation, along with the effects on exposed children’s development. They examined serum and breast milk concentrations of various medications in 60 mothers, observing changes over time and analyzing newborns’ development in the first year. Key findings include a decrease in serum concentrations of certain drugs during pregnancy, variable breast milk penetration ratios for different medications, and no clinically significant differences in birth outcomes or development between exposed and nonexposed children. The study highlights the importance of balancing risks and benefits in peripartum psychotropic medication use and emphasizes the need for continuous therapeutic drug monitoring to optimize dosing during pregnancy and lactation [173].

Yamamoto et al. investigated the association between NAS and plasma alprazolam concentration in a neonate born to a mother taking alprazolam throughout pregnancy. Symptoms such as apnea and vomiting were observed in the neonate, with the plasma alprazolam concentration gradually decreasing over three days. PBPK modeling and simulation supported toxicological assessment in special populations like pregnant women and neonates, suggesting a useful methodology for such evaluations, especially when clinical data are lacking [174]. Several studies have investigated the association between benzodiazepine or Z-hypnotic use during early pregnancy and adverse neonatal outcomes such as stillbirth, preterm birth, and small for gestational age (SGA). A study conducted in Taiwan aimed to evaluate this association using a nationwide, population-based cohort study. Researchers analyzed data from various sources, including birth certificates, health insurance records, and maternal and child health databases. The study cohort included singleton pregnancies of females aged 15–50 years who gave birth between 2004 and 2018. The findings revealed that early pregnancy exposure to benzodiazepines or Z-hypnotics was associated with adverse neonatal outcomes, with increased risks of stillbirth, preterm birth, and SGA compared to non-exposure. However, after controlling for confounding factors, the association with stillbirth risk was attenuated, suggesting potential confounding by indication. Despite this attenuation, the association remained significant for preterm birth and SGA [175]. Additional analyses using sibling controls and paternal negative control designs supported the findings, indicating that early exposure to benzodiazepines or Z-hypnotics was not associated with an increased risk of stillbirth and preterm birth but remained significantly associated with SGA. Late pregnancy exposure to these medications was also associated with substantially increased risks of stillbirth and preterm birth [176]. Combining PK data and PBPK modeling could enhance our understanding of drug safety during pregnancy, validate observational findings, and guide clinical practice. This would allow us to compare simulated exposure levels with observed data from pregnant individuals who took the medication, assess whether the PBPK model predicts adverse outcomes consistent with the observational findings, and consider individualized treatment approaches based on predicted exposure and risk profiles. Lastly, a population-based cohort study in South Korea assessed the association between first-trimester benzodiazepine use and the risk of major congenital malformations in offspring. The findings revealed a small increased risk of overall malformations and heart defects associated with first-trimester benzodiazepine exposure, particularly at higher daily doses. Considering the benefits of benzodiazepines for their major indications, the study recommended prescribing the lowest effective dosage during pregnancy to minimize potential risks [177]. Collectively, these studies provide valuable insights into the pharmacokinetic and clinical implications of benzodiazepine use during pregnancy. While benzodiazepines are effective for treating various medical conditions, clinicians should exercise caution when prescribing them to pregnant women, especially during late pregnancy, to mitigate potential risks to neonatal health. Further research is warranted to better understand the long-term effects of benzodiazepine exposure during pregnancy on offspring health.

#### 3.3.2. Real-World Evidence to Improve Decision Making of Best Therapeutic Approaches in Psychotropic Drugs in Pregnant Women

Real-world evidence provides valuable insights into the use of psychotropic drugs in pregnant women, aiding in decision making for the best therapeutic approaches. Studies have shown an increasing trend in exposure rates of psychotropic drugs among pregnant women over the last two decades, with common drugs being antipsychotics and antidepressants. Research highlights the importance of understanding prescribing patterns, exposure rates, and acceptance of different classes of psychotropic drugs during pregnancy to enhance decision making. Additionally, the use of multiple psychotropic medications during pregnancy, known as polypharmacy, has been observed, emphasizing the need for a comprehensive understanding of drug exposures and their implications [178].

Real-world data also shed light on the complexities of psychotropic medication use during pregnancy, such as the association between prenatal drug use and psychiatric admissions, as well as the evolving trends in drug utilization over time. Studies have indicated a reduction in psychotropic drug use across pregnancy, with variations in prescribing patterns compared to treatment guidelines, underscoring the necessity for a thorough review of practices. Furthermore, the research emphasizes the importance of considering the underlying indications for medication use and potential risks associated with exposure to multiple classes of psychotropic drugs during pregnancy [179,180]. Real-world evidence plays a crucial role in guiding healthcare professionals and pregnant women in making informed decisions regarding the use of psychotropic drugs during pregnancy [181]. By analyzing exposure rates, prescribing patterns, and outcomes associated with different classes of psychotropic medications, healthcare providers can optimize therapeutic approaches and ensure the safety and well-being of both the mother and the developing fetus [182].

## 4. Implications on Neonatal Health Outcomes

The use of psychotropic medications during pregnancy, including antidepressants, antipsychotics, stimulants, and benzodiazepines, can have implications for neonatal health outcomes. Exposure to these medications in utero can lead to various effects on newborns, such as Poor Neonatal Adaptation (PNA) and withdrawal symptoms. Neonates exposed to psychotropic drugs may develop symptoms like mild neurologic, autonomic, respiratory, and gastrointestinal abnormalities, which typically appear within 48 h after birth and last for 2–6 days [183]. Despite these potential issues, breastfeeding post-exposure to certain psychotropic medications like selective serotonin reuptake inhibitors (SSRIs), mirtazapine, or venlafaxine may offer a protective effect against the development of PNA. This protective mechanism is believed to be due to the continued, albeit lower, exposure to the medication through breast milk, which can help mitigate withdrawal symptoms and support neonatal adaptation [37,184]. Continuous observation of the mother and child at the maternity ward is recommended to detect potential PNA symptoms. Severe cases may require admission to the Neonatal Care Unit (NCU). Dr. Loretta Finnegan, in the 1970s, recognized the need for a standardized method to assess neonatal withdrawal symptoms and developed a scoring system based on clinical observations [185]. The Finnegan Neonatal Abstinence Syndrome (NAS) Scoring System is a tool used to assess and manage withdrawal symptoms in newborns who have been exposed to opioids or other substances in utero. The Finnegan NAS Scoring System helps healthcare providers evaluate and quantify the severity of withdrawal symptoms in newborns. It guides treatment decisions by identifying infants who require pharmacological intervention and monitoring their response to treatment [186]. There are some important challenges and considerations to note about this system: (1) inter-rater reliability: there can be variability in scoring between different healthcare providers, emphasizing the importance of standardized training; (2) non-specificity: some symptoms of NAS can overlap with other medical conditions, requiring careful differential diagnosis; (3) treatment guidelines: the Finnegan NAS Scoring System informs treatment decisions, but clinical judgment and individualized care are also crucial in managing infants with NAS [186,187]. This standardized tool plays a vital role in the assessment and management of neonatal withdrawal symptoms. It helps healthcare providers monitor newborns exposed to substances in utero, ensuring timely intervention and improving outcomes for these vulnerable infants. While most cases of PNA are mild and self-limiting, severe cases may necessitate phenobarbital treatment. Further research is needed to understand the long-term effects of PNA on child development.

In addition to breastfeeding, there are other approaches to consider for managing neonatal exposure to psychotropics. For medications with a shorter half-life, gradual tapering before delivery can reduce the neonate’s exposure. This approach minimizes the abrupt cessation of medication and helps prevent withdrawal symptoms in the newborn. Administering medications immediately after breastfeeding can help reduce the peak concentration in breast milk. This ensures that the neonate receives a lower dose during breastfeeding. Nonetheless, it is ideal to opt for psychotropics with minimal transfer into breast milk. Moreover, it is recommended to regularly assess the neonate for any adverse effects or withdrawal symptoms. If symptoms are observed, adjust the medication regimen or consider alternative treatments.

Benzodiazepines can lead to symptoms like floppy infant syndrome and PNA in newborns, especially when exposed during the last trimester of pregnancy. Factors influencing the development of these symptoms include the type of benzodiazepine, dose, duration of drug ingestion, and metabolism by the infant. High dosages and long half-life benzodiazepines pose a higher risk of toxicity, while those with short half-lives increase the risk of withdrawal symptoms. Symptoms of floppy infant syndrome and PNA can persist for hours to days after birth [188]. Benzodiazepines can transfer from the mother’s bloodstream into breast milk. The extent of transfer depends on factors such as the specific benzodiazepine, its half-life, and the timing of administration relative to breastfeeding. If a breastfeeding mother takes benzodiazepines, her infant may experience increased drowsiness or sedation. Short-term use of benzodiazepines (e.g., for acute anxiety or panic attacks) is generally considered safer during breastfeeding. The drug’s half-life plays a role; shorter-acting benzodiazepines are preferable [189,190].

The use of multiple classes of psychotropic medications during pregnancy can increase the risks to the infant. Studies have shown higher risks of congenital heart defects among infants exposed to both SSRI antidepressants and benzodiazepines during pregnancy. Understanding the complete picture of exposures to psychotropic medicines during pregnancy is crucial for assessing the potential impact on neonatal health outcomes. Parpinel et al. investigated the outcomes of pregnancy in women with depressive disorders compared to healthy controls. They found that depressed women had higher rates of cesarean section, preterm delivery, induction of labor, and small-for-gestational-age (SGA) babies, along with lower neonatal weights and 5 min APGAR scores. However, those treated with psychotropic drugs showed a reduced rate of cesarean section compared to untreated patients, with no significant differences in other complications observed between treated and untreated women [191]. Brumbaugh et al. focused on the associations between antidepressant exposure during the third trimester of pregnancy and the risk of poor neonatal adaptation (PNA). They found that infants exposed to serotonergic antidepressants, especially SSRIs at higher doses, had an increased risk of PNA compared to those exposed to standard doses or other antidepressants. Additionally, maternal anxiety disorders were identified as potential risk factors for PNA [183]. Finally, Tharp et al. conducted a retrospective cohort study to determine if lack of exposure to individual antidepressants at certain times during pregnancy improved maternal and infant outcomes. They found that for most antidepressants, lack of exposure early or late in pregnancy did not significantly change newborn outcomes. However, for bupropion and escitalopram, lack of exposure in the third trimester was associated with lower rates of neonatal intensive care unit (NICU) admission or adaptation syndrome in the newborn [192]. These studies provide valuable insights into the complex relationship between depressive disorders, pharmacological treatment during pregnancy, and maternal and neonatal outcomes, highlighting the need for individualized care and informed discussions between pregnant women and healthcare providers regarding the risks and benefits of antidepressant use during pregnancy.

It is crucial to study confounding factors when examining infant outcomes with exposure to psychotropic medications during pregnancy. Many studies are confounded by the illnesses, behaviors, and other risk factors associated with psychiatric illness in the mother. For instance, women with psychiatric disorders who discontinue medication during pregnancy have a very high risk of relapse, which can negatively impact the infant. Untreated maternal depression and anxiety can also lead to pregnancy complications, irritability and lethargy in infants, and attachment problems between mother and child. To properly assess the risks of psychotropic medications, it is important to compare outcomes between women exposed to the medications during pregnancy and those with past exposure, as well as those with psychiatric disorders who never used the medications. Sibling-matched analyses can also help control for confounding factors. Factors, like the strength and direction of the exposure–outcome association, replication across studies, specificity of the association, temporal and dose–response relationships, and biological plausibility, should be considered. Failure to consider confounding variables can result in misleading interpretations of study results. Healthcare providers rely on evidence-based research to make informed decisions about the management of psychiatric disorders during pregnancy. Understanding the potential impact of confounding variables allows clinicians to critically evaluate study findings and weigh the risks and benefits of psychotropic medication use in pregnant patients based on more accurate and comprehensive evidence [193,194].

## 5. Future Directions and Challenges in Antipsychotic Therapy for Pregnant Women

Advancements have been made in the field of psychotropic therapy for pregnant women, yet there are important considerations and areas for improvement. Prospective studies examining the safety and efficacy of psychotropic drugs during pregnancy are crucial for providing evidence-based guidelines. Long-term follow-up studies are also necessary to assess the impact of these medications on both the mother and the developing child. Pregnancy is a unique period, and each woman’s response to psychotropic medications can vary. Tailored treatment plans based on individual risk factors, psychiatric history, and specific symptoms are necessary to ensure optimal outcomes. Clinicians must carefully consider maternal and fetal health when prescribing these drugs, striking a balance between potential benefits and risks.

Integrating psychosocial interventions alongside pharmacotherapy can enhance treatment outcomes. Collaborative care models involving psychiatrists, obstetricians, and other healthcare providers are instrumental in optimizing treatment strategies. Patient education is vital, with clear information about the risks and benefits of psychotropic medications needed to facilitate informed decision making. Balancing the potential risks of untreated psychiatric illness with the risks of medication exposure is challenging. Shared decision making between the woman and her healthcare team is crucial, particularly for severe conditions where the benefits of treatment may outweigh potential risks. Regular monitoring during pregnancy and the postpartum period is essential, with close attention needed to pharmacokinetic changes and neonatal outcomes.

The impact of psychotropic exposure on neonatal outcomes remains an area of interest; taking psychotropic medications requires careful evaluation, with clinicians weighing the benefits against potential risks associated with drug exposure through breast milk. Collaborative efforts among researchers, clinicians, and regulatory agencies are essential for advancing knowledge in this field. Funding support for large-scale studies and international registries tracking outcomes of pregnant women exposed to psychotropic drugs can provide valuable data. Additionally, reducing stigma around mental health during pregnancy is vital to ensure women feel comfortable seeking help and discussing treatment options. Community awareness campaigns can play a key role in promoting understanding and acceptance.

## Figures and Tables

**Table 1:** Consideration to enhance therapeutic monitoring in pregnant women.

Category	Description	Examples of Medications	Challenges and Considerations	Technologies and Innovations
Importance of TDM in Pregnancy	Monitoring is crucial due to physiological changes that affect drug pharmacokinetics.	Antiepileptics, Antidepressants, Antibiotics	Changes in drug disposition, treatment efficacy, toxicity.	Physiologically based pharmacokinetic models.
Medications of Interest	Drugs requiring careful dose adjustments due to individual variability and narrow therapeutic indices.	Phenytoin, Carbamazepine, Antipsychotics, Antidepressants.	Need for specific clinical studies in pregnant women, ethical challenges.	Nucleic acid assays, wearable technologies.
Monitoring Technologies	Advanced tools for accurately and real-time monitoring plasma drug levels.	CGM for glucose, Bio-nanochip devices, DBS.	Cost-effectiveness, accessibility, integration with healthcare systems.	Smartphone integration, genomic and proteomic microarrays.
Recent Innovations	Advances improving the sensitivity and usability of monitoring technologies.	Enzyme-based biosensors, wearable for L-Dopa.	Long-term stability, accuracy in real-world environments.	Smartphone-integrated sensors, paper-based lateral flow devices.
Clinical and Economic Impact	Evaluation of TDM effectiveness in improving maternal and fetal outcomes and reducing perinatal complications.	Anticonvulsant monitoring, gestational diabetes management.	Need for more research on cost-effectiveness and impact on clinical practice.	Development of automated, low-cost PoC systems.
Considerations in Breastfeeding	Importance of monitoring medication levels during breastfeeding to minimize infant exposure.	Antidepressants, Antipsychotics, Antibiotics.	Monitoring of mother and baby, dose adjustments.	Home DBS collection, real-time analysis.
Implementation Challenges	Barriers to widespread adoption of TDM, including complexity, costs, and clinical consensus.	Antiepileptics, Psychotropic medications.	Lack of clear cost-effectiveness data, limited funding.	PoC technologies, saliva monitoring with bio-nanochips.
Accessibility and Usability	Perspectives of pregnant women and healthcare providers on the accessibility and usability of mobile technologies for monitoring during pregnancy.	Monitoring apps, wearable devices.	Usability, additional workload for healthcare providers.	User feedback, technological acceptance studies.

## Data Availability

Not applicable.
